# Molecular epidemiology and surveillance of circulating rotavirus among children with gastroenteritis in Bangladesh during 2014–2019

**DOI:** 10.1371/journal.pone.0242813

**Published:** 2020-11-30

**Authors:** Shuvra Kanti Dey, Nadim Sharif, Omar Sadi Sarkar, Mithun Kumar Sarkar, Ali Azam Talukder, Tung Phan, Hiroshi Ushijima

**Affiliations:** 1 Department of Microbiology, Jahangirnagar University, Savar, Dhaka, Bangladesh; 2 University of Louisville, Louisville, Kentucky, United States of America; 3 University of Pittsburgh Medical Center, Pittsburgh, Pennsylvania, United States of America; 4 Division of Microbiology, Department of Pathology and Microbiology, Nihon University, Tokyo, Japan; Defense Threat Reduction Agency, UNITED STATES

## Abstract

Acute gastroenteritis is one of the major health problems in children aged <5 years around the world. Rotavirus A (RVA) is an important pathogen of acute gastroenteritis. The burden of rotavirus disease in the pediatric population is still high in Bangladesh. This study investigated the prevalence of group A, B, and C rotavirus (RAV, RBV, RCV), norovirus, adenovirus (AdV) and human bocavirus (HBoV) infections in children with acute gastroenteritis in Bangladesh from February 2014 to January 2019. A total of 574 fecal specimens collected from children with diarrhea in Bangladesh during the period of February 2014-January 2019 were examined for RAV, RBV and RCV by reverse transcriptase- multiplex polymerase chain reaction (RT- multiplex PCR). RAV was further characterized to G-typing and P-typing by RT-multiplex PCR and sequencing method. It was found that 24.4% (140 of 574) fecal specimens were positive for RVA followed by AdV of 4.5%. RBV and RCV could not be detected in this study. Genotype G1P[8] was the most prevalent (43%), followed by G2P[4] (18%), and G9P[8] (3%). Among other genotypes, G9P[4] was most frequent (12%), followed by G1P[6] (11%), G9P[6] (3%), and G11P[25] (3%). We found that 7% RVA were nontypeable. Mutations at antigenic regions of the VP7 gene were detected in G1P[8] and G2P[4] strains. Incidence of rotavirus infection had the highest peak (58.6%) during November to February with diarrhea (90.7%) as the most common symptom. Children aged 4–11 months had the highest rotavirus infection percentage (37.9%). By providing baseline data, this study helps to assess efficacy of currently available RVA vaccine. This study revealed a high RVA detection rate, supporting health authorities in planning strategies such as introduction of RVA vaccine in national immunization program to reduce the disease burden.

## Introduction

Acute gastroenteritis (AGE) is one of the major burden of diseases in children in the developing and developed countries [1, 2]. Viruses are the main pathogen of AGE in children worldwide [1, 3–5]. The incidence of pediatric AGE has been reduced in the developed countries in recent years [1]. However, virus associated pediatric AGE is still a major health burden in developing countries [1, 2, 8]. Among enteropathogenic viruses, rotavirus A (RVA) (the family *Reoviridae*) is the main causative agent of pediatric AGE [1–3]. It is estimated that about 3–5 billion cases of pediatric AGE occur every year around the world. Every year about 3 million children die of AGE worldwide [4–8]. Rotavirus A has been reported as the main pathogen from about one million deaths associated with pediatric AGE [4–8]. The incidence of rotavirus is estimated to be 10,000 cases per 100,000 children in children below five years in Bangladesh. About 27% of rotavirus infected children develop severe health conditions. Rotavirus associated mortality is estimated to be 2000 to 3000 per year in Bangladesh [9].

Rotavirus is a nonenveloped virus having a genome of segmented double stranded RNA (dsRNA) of about 18.55 kilo base pairs [10]. The genome encodes for six structural proteins called VP1 to VP6 and six nonstructural proteins called NSP1 to NSP6 [10]. There are ten species of the genus *Rotavirus*, referred to as A, B, C, D, E, F, G, H, I and J [11]. Among them, RVA is the main human pathogen and causes 90% of the diseases, while RVB and RVC are also found to be involved in human diseases [12]. RVA is further classified in two genotypes called as G genotype from VP7 or glycoprotein of the virus and P genotype from VP4 or protease [11, 13]. About 36 rotavirus G types and 51 rotavirus P types have been reported [14, 15]. The most common G types are G1-G4, G9 and G12, whereas P[4], P[6] and P[8] are the most common P genotypes worldwide [14–17]. The most commonly found genotype combination of RVA in human are G1P[8], G2P[4], G3P[8], G4P[8] and G9P[8] [16–18]. In Bangladesh, the genotypic distribution of rotavirus has changed over the years. Rotavirus genotype G1P[8], G2P[4], G3P[8], G4P[8] and G9P[8] have been reported from children with acute gastroenteritis in Bangladesh [16–21].

Four rotavirus A vaccines (RotaTeq-Merck, Rotarix- GSK, ROTAVAC, and ROTASIIL) have been prequalified by WHO. India, Vietnam and China have developed rotavirus A vaccine for their own market [19, 22]. Global rotavirus vaccines are available in Bangladesh market but the vaccination program has not been included in the national immunization program yet [9, 19]. Rotavirus vaccine coverage in children under five is high in Bangladesh. For rotavirus vaccine, DTP1 is 97%, DPT2 is 95.5% and DTP3 is 94% in Bangladesh. A recent Rotarix trial in Bangladesh has revealed that vaccine efficacy is decreasing due to the mutational and reassortment events of rotaviruses [9]. Molecular analysis and genetic characterization of rotavirus A will specify diversity that will be an important tool to evaluate vaccine efficacy [23, 24].

The main aim of this study is to characterize the genotypes of rotavirus A in Bangladesh. First objective of this study is to describe the age distribution, gender distribution, seasonal pattern and genotypic distribution of rotavirus A among children in Bangladesh. Second objective of this study is to describe intragenotypic heterogeneity of rotavirus G1 circulating in Bangladesh.

## Materials and methods

### Study population and fecal specimens

Between February 2014 and January 2019, 574 fecal specimens were collected from children with acute gastroenteritis from four clinics in three different regions (Chattogram, Savar and Sirajganj) in Bangladesh. Each clinic located in each region, except for Savar with two clinics. All children aged <15 years with viral gastroenteritis (clinically suspected) were enrolled in the study. Samples were collected from pediatric patients with three or more watery non-bloody stools/24 hours. Non-infectious bloody diarrheal cases were excluded following WHO case standard guideline. The ages ranged from 55 days to 15 years with a mean age of 24 months. One fecal specimen was collected from each patient. Informed consent of the parents was taken before sample collection. Ten percent suspension of the fecal specimens were made and centrifuged at 10000x g for 10 min. All the specimens were stored at -20°C until further analysis.

### Ethical approval

This study was ethically approved by the Biosafety, Biosecurity & Ethical Committee (BBEC) of Jahangirnagar University. Ethics committee approval number for this study is BBEC, JU/M 2020/(10)3.

### Viral RNA and DNA extraction

Fecal suspensions were thawed and centrifuged at 10000x g for 10 min. Viral RNA was extracted using 140 μL of the supernatant by SV Total RNA Isolation System (Promega kit) according to the manufacturer’s protocols (Promega, Madison, USA). Viral DNA for adenoviruses and human bocaviruses was extracted following the established protocols in Sharif et al. (2020).

### Complementary DNA (cDNA) synthesis by reverse transcription

For rotavirus and norovirus, cDNA was synthesized using Promega cDNA synthesis kit (Promega, Madison, USA). For reverse transcription (RT), 3 μL of extracted RNA (100 ng) was mixed with a reaction mixture, consisting of 1 μL of oligo dT primer (25 μg/ml) and 1 μL of nuclease free water in nanocentrifuge tube, kept at 70°C for 5 min and chilled for 5 min in ice. After that 4 μL of 5X reaction buffer, 2 μL of 5 mM MgCl_2_, 1 μL of 10 mM PCR Nucleotide Mix, 0.5 μL of ribonuclease inhibitor (1 u/ μL), 1 μL of reverse transcriptase, and 6.5 μL of nuclease free water were mixed for each sample in the same nanocentrifuge tube, containing 5 μL of previously chilled genomic RNA. Total volume of the mixture was 20 μL. The mixture was heated at 25°C for 5 min, 42°C for 60 min and 70°C for 15 min. The cDNA was prepared and stored at -20°C.

### Polymerase chain reaction (PCR)

For rotavirus A, two specific primers F-^1^GGCTTTAAAAGAGAGAATTTC^28^ and R-^373^ACTGATCCTGTTGGCCATCCTTT^395^ were used to amplify VP7 gene. For norovirus, two specific primers F-^5342^CGCCGTGGCTCCTGCTCT^5361^ and R-^5671^GTTCGCCATCACAAAAGATGTG^5653^ were used to amplify VP1 gene. For adenovirus, two specific primers AD1-F ^1834^TTCCCCATGGCICAYAACAC^1853^ and AD2-R ^2315^CCCTGGTAKCCRATRTTGTA^2296^ were used to amplify hexon gene. For human bocavirus, two specific primers AK-VP-F1 ^3274^CGCCGTGGCTCCTGCTCT^3291^ and AK-VP-R1 ^3860^TGTTCGCCATCACAAAAGATGTG^3876^ were used to amplify VP1 gene [25]. The PCR reaction was performed at 94°C for 3 min, followed by 35 cycles of denaturation at 94°C for 1 min, annealing at 55°C for 1 min and extension at 72°C for 1 min. Final extension was done at 72°C for 7 min and then held at 4°C. PCR reaction mixture contained 12.5 μL of the 2X master mix (GoTaq^®^Green Master Mix, Promega, USA), 1 μL of 1 μM forward (F) primer, 1 μL of 1 μM reverse (R) primer, 6.5 μL of nuclease free water, and 4 μL of template (200 ng). For rotavirus and norovirus, 4 μL cDNA (200 ng) was used as template. Total volume of the PCR reaction mixture was 25 μL. Previously sequenced samples were used as positive controls. The PCR was performed in 2720 Thermal Cycler (Applied Biosystems, USA) [25].

### Group A rotavirus G typing

Group A rotavirus G typing was conducted using the protocol from the previously described method [23]. Reverse transcription (RT) was performed for the full-length VP7 gene, and the first amplification was completed by using Beg9 and End9 primers. The second amplification was performed using the first PCR product as the template with G type-specific mixed primers 9T1-1, 9T1-2, 9T-3P, 9T-4, and 9T-B and forward primer 9con1 (9con1 is specific primer that is used to characterize G genotype). Amplicons of 158 bp, 224 bp, 466 bp, 403 bp, and 110 bp were specifically generated for G1, G2, G3, G4, and G9, respectively.

### Group A rotavirus P typing

Group A rotavirus P typing was conducted using the protocol from the previously described method [23]. The first amplification of VP4 gene was completed by using Con2 and Con3 primers by RT-PCR. The second amplification was performed by using a mixture of primers 1T-1, 2T-1, 3T-1, 4T-1, 5T-1, and Con3 for the identification of P[8], P[4], P[6], P[9], and P[10], with amplicons of 346 bp, 484 bp, 268 bp, 392 bp, and 584 bp, respectively.

### Agarose gel electrophoresis

The amplicons were electrophoresed using 1.5% agarose gel. The gel run was continued for 25 min horizontally. Separated amplicons were visualized using UV spectrophotometer (SPECORD-205, Analytik-Jena, Germany) and photographed in the Gel doc system (BioRad, USA).

### Nucleotide sequence analysis

The nucleotide sequences of PCR amplicons positive for rotavirus A, norovirus, human bocavirus and adenovirus were determined with the Big-Dye terminator cycle sequencing kit and an ABI Prism 310 Genetic Analyzer (Applied Biosystems Inc. Foster City, CA) [25]. The sequences were analyzed using Chromas 2.6.5 (Technelysium, Helensvale, Australia). The sequence data were converted into FASTA format. Sequence homology was confirmed using the BLASTn (https://blast.ncbi.nlm.nih.gov/Blast.cgi) program. Multiple sequence alignment was performed in the BioEdit 7.2.6 software using the ClustalW Multiple Alignment algorithm [26]. The similarity matrix was computed using the Maximum Composite Likelihood model. The nucleotide sequence data of all Bangladeshi RVA had been submitted to the GenBank database.

### Phylogenetic analysis

Phylogenetic and evolutionary relationship analysis of RVA was conducted using VP7 gene and the reference sequences by the MEGA X software [27]. Phylogenetic trees with 1000 bootstrap replicates of the nucleotide alignment datasets were generated. For calculating the genetic distance, Kaimura-2 parameter model was used. Maximum Composite Likelihood (MCL) method was used for phylogenetic relationship analysis [28]. The reference strains used for RAV phylogenetic analysis are provided in the S1 Chart.

### Nucleotide sequence submission

The nucleotide sequence data used in this study had been submitted to the GenBank database and had been assigned accession number MN202241 and MT310555-MT310574.

### Statistical analysis

Statistical analysis was conducted using IBM^®^ SPSS^®^ Statistics for windows, version 23.0 (IBM Corp. Armonk, New York, USA) software package. Prevalence of diarrheal pathogens were detected by dividing number of cases by total number of study children during the study period. Statistical analysis for phylogenetic data was calculated by kimura-2-parameter model in the MEGA X software [27, 28].

## Results

### Demographic characteristics of children with acute gastroenteritis

Demographic characteristics of 574 pediatric patients with acute gastroenteritis were analyzed. Among the pediatric patients, 55.6% (319 of 574) were male and 44.4% (255 of 574) were female. The study population was distributed into six age groups from 1 month to 60 months (1–3, 4–11, 12–23, 24–35, 36–47 and 48–60). Most of the patients (22.8%) belonged to 4–11 months group followed by other five age groups (Table 1). Among the pediatric patients, 69.2% (397 of 574) received primary treatment and fluid replacement, and 30.8% (177 of 574) didn’t receive any treatment at home.

**Table 1:** Demographic characteristics of patients who were enrolled in this study during 2014–2019 in Bangladesh.

Factors	Group	Percentage
**Gender**	Male	55.6% (319 of 574)
	Female	44.4% (255 of 574)
**Age group (months)**	1–3	14.9% (86 of 574)
	4–11	22.8% (131 of 574)
	12–23	21.6% (124 of 574)
	24–35	15.3% (88 of 574)
	36–47	12.5% (72 of 574)
	48–60	12.7% (73 of 574)
**Treatment conditions**	Received at home	69.2% (397 of 574)
	At hospital	30.8% (177 of 574)

### Detection of viruses associated with acute gastroenteritis in children

Among 574 fecal specimens, 36.8% (211 of 574) were positive for viral infection. RV was the most prevalent (24.4%, 140 of 574) followed by NoV (4.9%, 28 of 574), AdV (4.2%, 24 of 574) and HBoV (3.3%, 19 of 574), respectively [25]. About 63.2% (363 of 574) of the samples were tested negative for diarrheal viruses.

### Coinfection of children with acute gastroenteritis

Frequencies of both monoinfection and coinfection of pediatric patients were determined. Infection of viruses was found in 75.4% (159 of 211) of the cases. Coinfection between viruses was found in 24.6% (52 of 211) of cases. Dual infection between RV and NoV accounted for most of the cases (10) of coinfection, followed by RV-AdV (6), RV-HBoV (4), NoV-AdV (3), NoV-HBoV (2) and AdV-HBoV (1).

### Age and gender distribution of rotavirus among children with acute gastroenteritis

Viruses associated with acute gastroenteritis were detected in every group aged <60 months. Rotavirus was most prevalent (37.9%, 53 of 140) among children aged 4–11 months, followed by 12–23 months (22.1%, 31 of 140), 1–3 months (15.7%, 22 of 140), 24–35 months (15%, 21 of 140), 48–60 months (5%, 7 of 140), and 36–47 months (4.3% 6 of 140). The distribution of gender in rotavirus positive cases were 62.1% (87 of 140) in male, and 37.9% (53 of 140) in female. The ratio between rotavirus infected male and female was 1.6:1, but it was not significantly different.

### Clinical features of acute gastroenteritis associated with rotavirus infection

**Fig 1:**
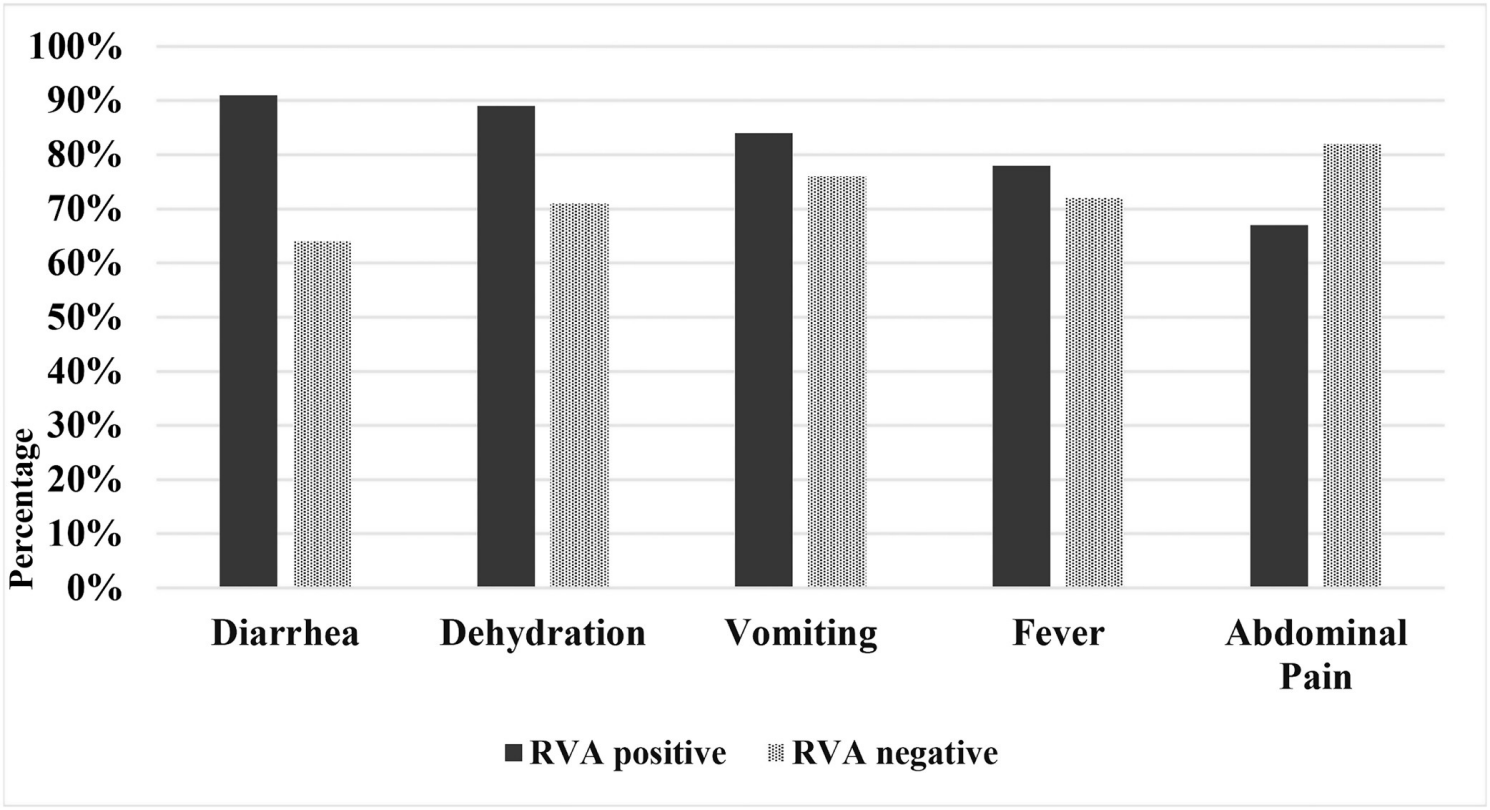
Comparison of clinical features between rotavirus positive children and rotavirus negative children during 2014–2019 in Bangladesh.

Viruses associated with acute gastroenteritis were found to be involved with various clinical complications of the pediatric population. Among rotavirus infected patients, diarrhea (90.7%) was the most common, followed by dehydration (88.7%), vomiting (84.3%), fever (77.8%) and abdominal pain (67.1%) (Fig 1). Whereas diarrhea (85.7%) was the most frequent in the norovirus infected children, vomiting (87.5%) was the most common in the adenovirus infected children, and dehydration (94.7%) was the most common in the human bocavirus infected children.

### Seasonal pattern of rotavirus infection

**Fig 2:**
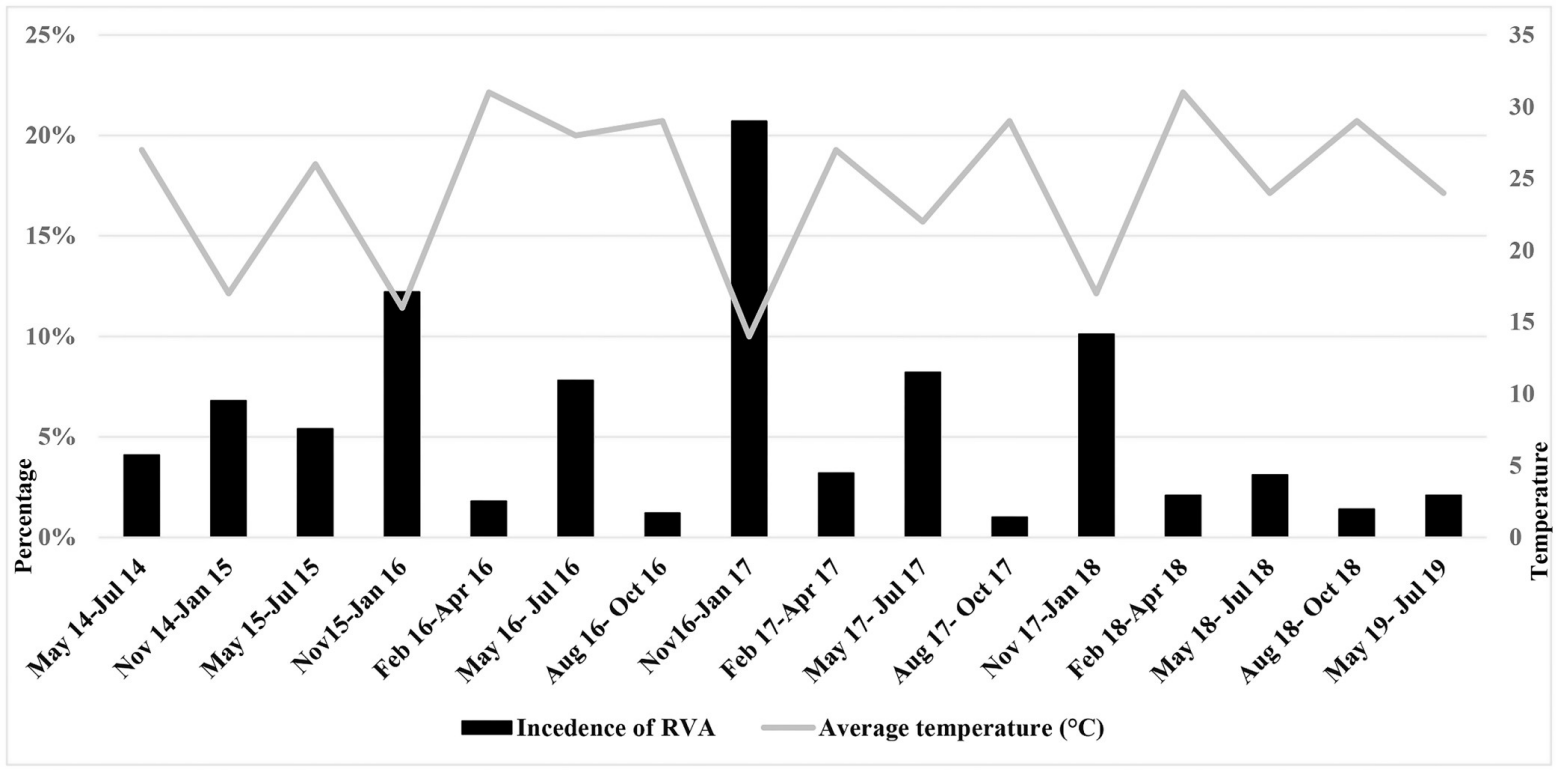
Seasonal pattern of rotavirus infection in infants and children with acute gastroenteritis along with correlation of rotavirus incidence with average temperature in Bangladesh during 2014–2019.

Rotavirus infection was detected all year round. But majority of rotavirus infection cases (58.6%) were found during the winter season (Nov-Jan), followed by 30.7% in the rainy season (May-Jul), 7.1% in the spring season (Feb-Apr), and 3.6% in the autumn season (Aug-Oct), respectively. During the winter season the average temperature was around 17°C. The incidence of rotavirus had a peak at a lower temperature in Bangladesh (Fig 2).

### Distribution of G and P genotypes of group A rotavirus

Among the G genotypes, G1 was the most predominant (57%), followed by G2 (20%), G9 (20%) and G11 (3%). Four P genotypes were detected during 2014 to 2019 in Bangladesh. Rotavirus genotype P[8] was the most frequent (46%), followed by P[4] (30%), P[6] (14%) and P[25] (3%). In combination of rotavirus G and P genotype, great diversity was detected. Rotavirus genotype combination G1P[8] was the most prevalent (43%), followed by G2P[4] (18%), G9P[4] (12%), G1P[6] (11%), G9P[8] (3%), G9P[6] (3%) and G11P[25] (3%), respectively. 7% rotavirus was nontypeable (Table 2).

**Table 2:** Distribution of group A rotavirus G and P genotypes among children with acute gastroenteritis in Bangladesh during 2014–2019.

G genotype	P genotype				Nontypable	Total of G and P combination (%)
	P[8]	P[4]	P[6]	P[25]		
G1	60 (43%)	-	**16 (11%)**^a^	-	**4 (3%)** ^a^	80 (57)
G2	-	25 (18%)	-	-	**3 (2%)** ^a^	28 (20)
G9	4 (3%)	17 (12%)	**4 (3%)** ^a^	-	**3 (2%)** ^a^	28 (20)
G11	-	-	-	**4 (3%)** ^a^	-	4 (3)
Total	64 (46%)	42 (30%)	**20 (14%)** ^a^	4 (3%)	**10 (7%)** ^a^	140 (100)

^a^Unusual G/P combinations are indicated in bold.

**Fig 3:**
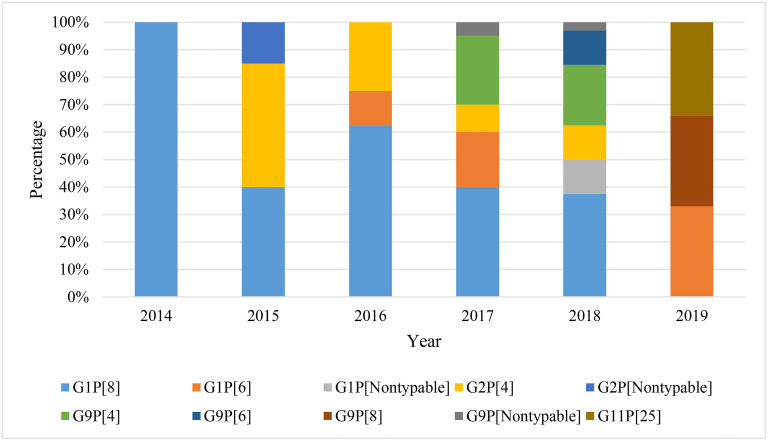
Yearly distribution of rotavirus genotype in children with acute gastroenteritis in Bangladesh during 2014–2019.

During the study period, rotavirus incidence was the highest in 2017 (28.6%), followed by 22.9% in 2018, 22.9% in 2016, 14.3% in 2015, 8.6% in 2019 and 2.9% in 2014. Genotype G1P[8] was detected in every year, but the highest incidence (33.3%) was found in 2016, followed by 26.7% in 2017, 20% in 2018, 13.3% in 2015 and 6.7% in 2014, respectively. Genotype G2P[4] was detected in the highest percentage (36%) in 2015, followed by 32% in 2016, 16% in 2017 and 16% in 2018, respectively. However, genotype G9P[4] was detected in the highest percentage (59%) in 2017. Genotype G1P[6] was detected in highest percentage (50%) in 2017, followed by 25% in 2016 and 25% in 2019, respectively. Genotype G1P[8] and G2P[4] were detected during 2014–2018, but none were detected in 2019. About 40% of nontypeable rotavirus was detected in 2018 (Fig 3). Of note, 51.4% rotavirus were detected from Savar region followed by 37.4% from Chattogram and 11.2% from Sirajganj.

### Nucleotide sequence and phylogenetic analysis of group a rotavirus

**Fig 4:**
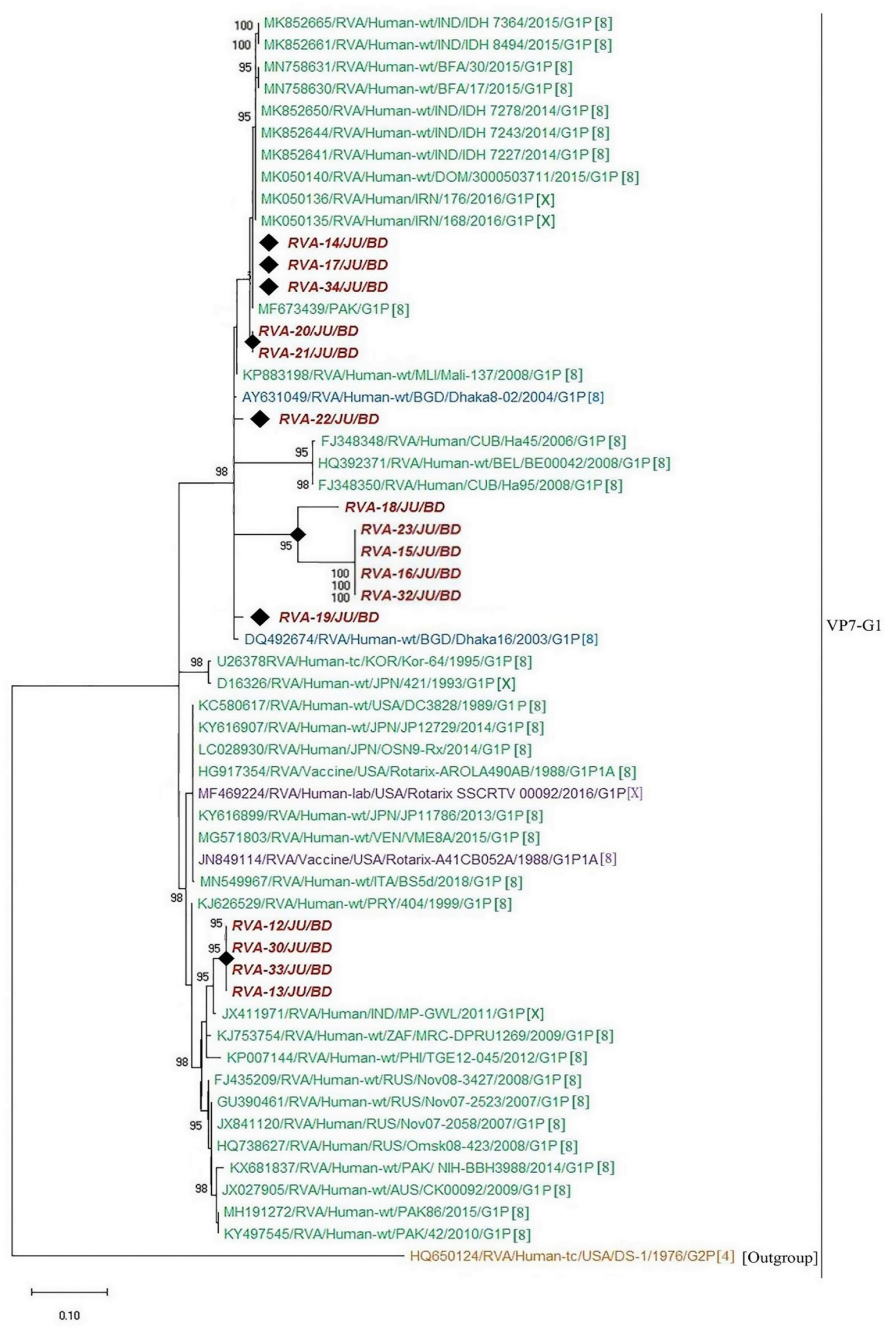
Maximum likelihood phylogenetic tree constructed from the nucleotide sequences of G1-VP7 strains and representative RVA strains with kimura-2-parameter model in MEGA X. Bootstrap values <60 are not shown. RVA strains sequenced in this study are represented by the red color in italic bold letter. Bangladeshi strains reported in the previous studies are shown in blue. The vaccine strains are represented by purple color, while green shading represent strains isolated all over the world. The RVA strains sequenced in this study are represented by their ID and reference strains obtained from GenBank database are represented by Accession number, Strain Name, Country and year of Isolation. Scale bar: 0.10 substitutions per nucleotide.

**Fig 5:**
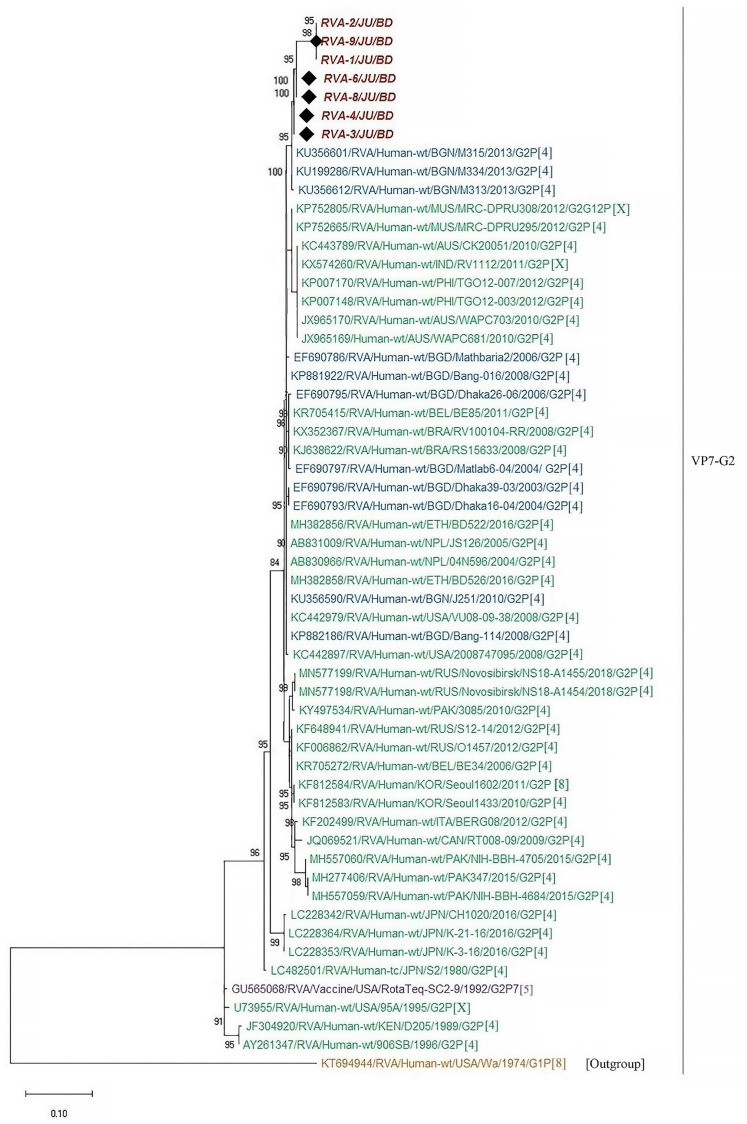
Maximum likelihood phylogenetic tree constructed from the nucleotide sequences of G2-VP7 strains and representative RVA strains with kimura-2-parameter model in mega program 10.0. Bootstrap values <60 are not shown. RVA strains sequenced in this study are represented by the red color in italic bold letter. Bangladeshi strains reported in the previous studies are shown in blue. The vaccine strains are represented by purple color, while green shading represent strains isolated all over the world. The RVA strains sequenced in this study are represented by their ID and reference strains obtained from GenBank database are represented by Accession number, Strain Name, Country and year of Isolation. Scale bar: 0.10 substitutions per nucleotide.

**Fig 6:**
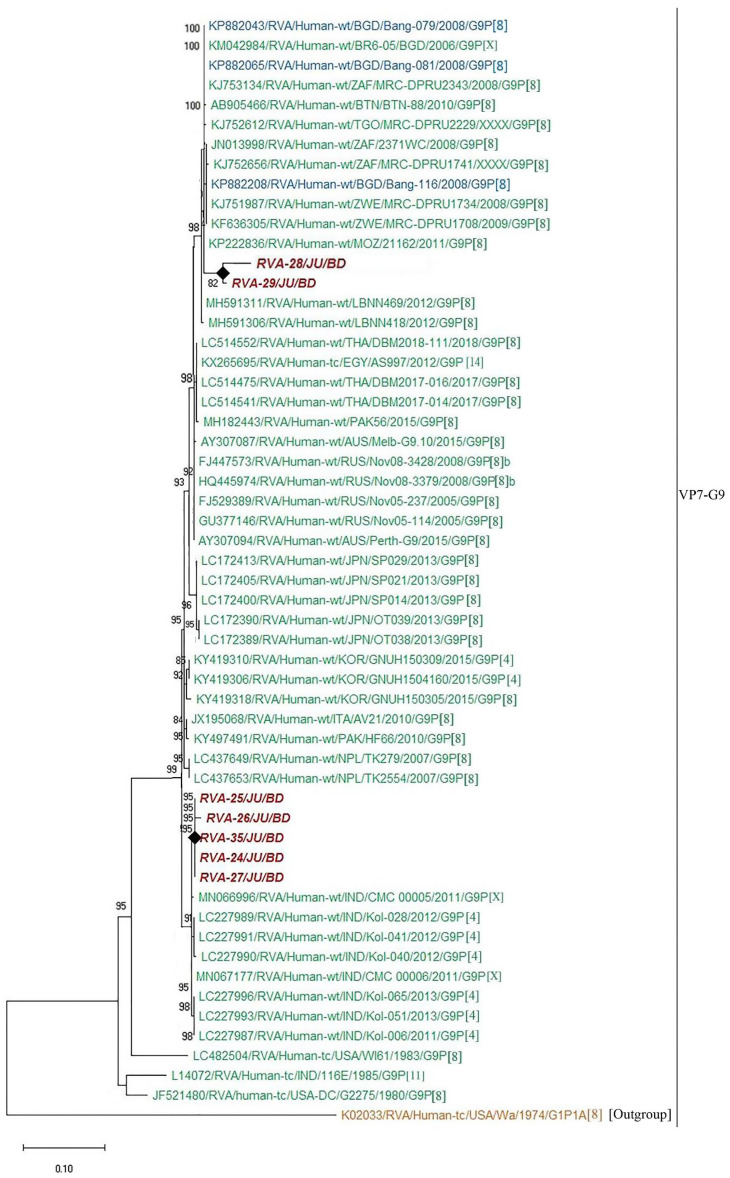
Maximum likelihood phylogenetic tree constructed from the nucleotide sequences of G9-VP7 strains and representative RVA strains with kimura-2-parameter model in MEGA X. Bootstrap values <60 are not shown. RVA strains sequenced in this study are represented by the red color in italic bold letter. Bangladeshi strains reported in the previous studies are shown in blue, **whereas** green shading represent strains isolated all over the world. The RVA strains sequenced in this study are represented by their ID and reference strains obtained from GenBank database are represented by Accession number, Strain Name, Country and year of Isolation. Scale bar: 0.10 substitutions per nucleotide.

**Fig 7:**
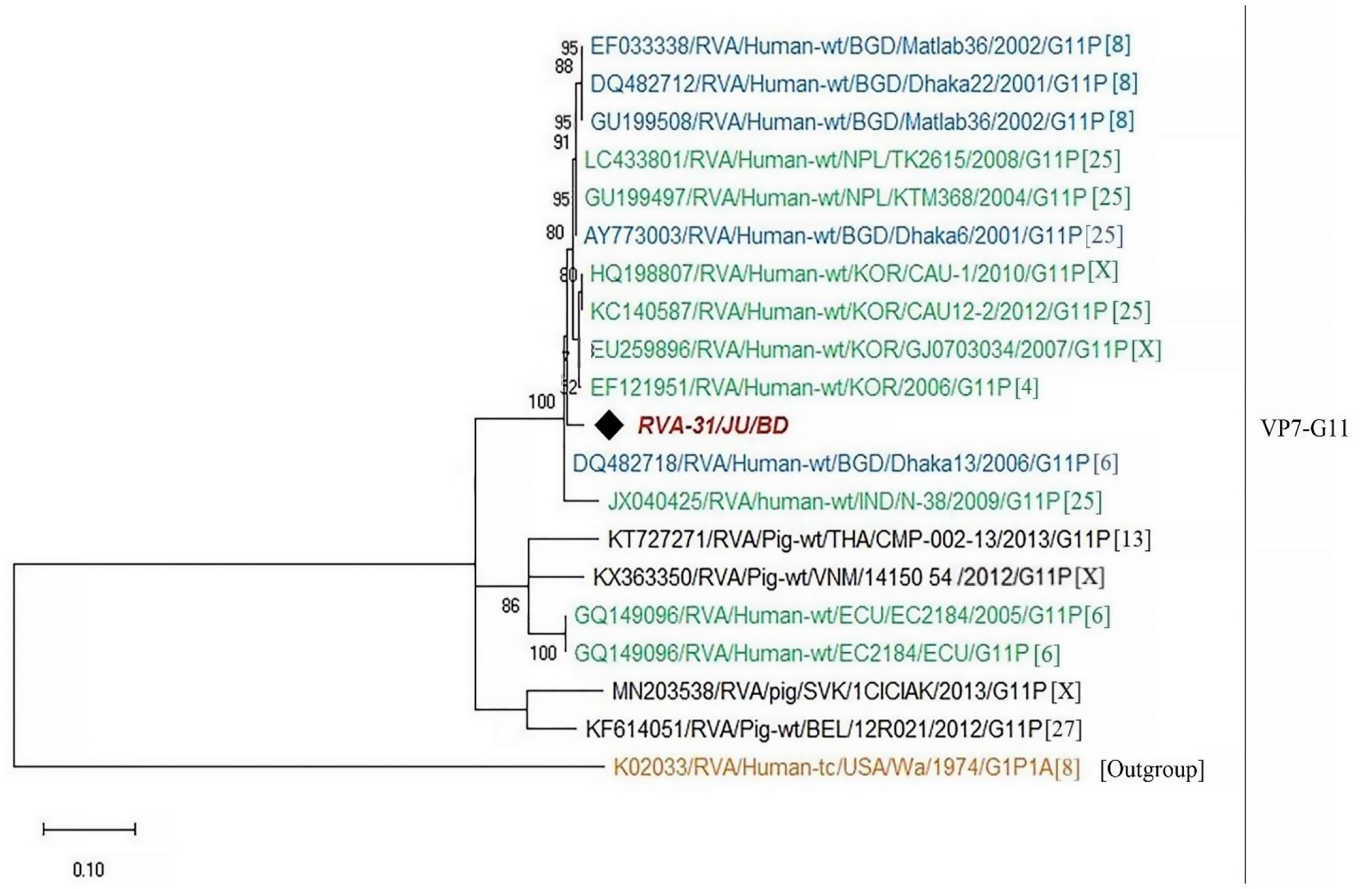
Maximum likelihood phylogenetic tree constructed from the nucleotide sequences of G11-VP7 strains and representative RVA strains with kimura-2-parameter model in MEGA X. Bootstrap values <60 are not shown. RVA strains sequenced in this study are represented by the red color in italic bold letter. Bangladeshi strains reported in the previous studies are shown in blue, while green shading represent strains isolated all over the world and black color represented nonhuman primate RVA strains. The RVA strains sequenced in this study are represented by their ID and reference strains obtained from GenBank database are represented by Accession number, Strain Name, Country and year of Isolation. Scale bar: 0.10 substitutions per nucleotide.

In order to understand the molecular epidemiology of rotavirus A in Bangladesh, sequence analysis of VP7 gene were conducted. A total of thirty-five representative sequences of rotavirus A were used for the phylogenetic analysis. Genotype G1P[8] was the most prevalent (45.7%), followed by G2P[4] (20%), G9P[4] (14.3%), G1P[6] (11.4%), G9P[8] (2.9%), G9P[6] (2.9%) and G11P[25] (2.9%), respectively. RVA genotype G1P[8] in Bangladesh during 2014–2019 were closely related with G1P[8] of India, Mali, Russia, Pakistan, Cuba, Iran and previous strains of Bangladeshi rotavirus G1P[8] (Fig 4). Further, genotype G2P[4] detected during 2014–2019 in Bangladesh clustered with each other and previously reported G2P[4] of Bangladesh, Mauritius and Australia (Fig 5). We also confirmed the genotypic relationship of G9 and G11 detected in Bangladesh with other circulating rotaviruses worldwide. Study genotype G9P[4] clustered with G9P[4] strain of India and study genotype G9P[8] was closely related with previously reported G9P[8] of Bangladesh, Mozambique and Thailand (Fig 6). Study genotype G11P[25] clustered with the G11P[25] of Korea, G11P[4] of Korea and G11P[6] of Bangladesh (Fig 7).

### Distribution of G1 genotypes into unique sublineages

**Fig 8:**
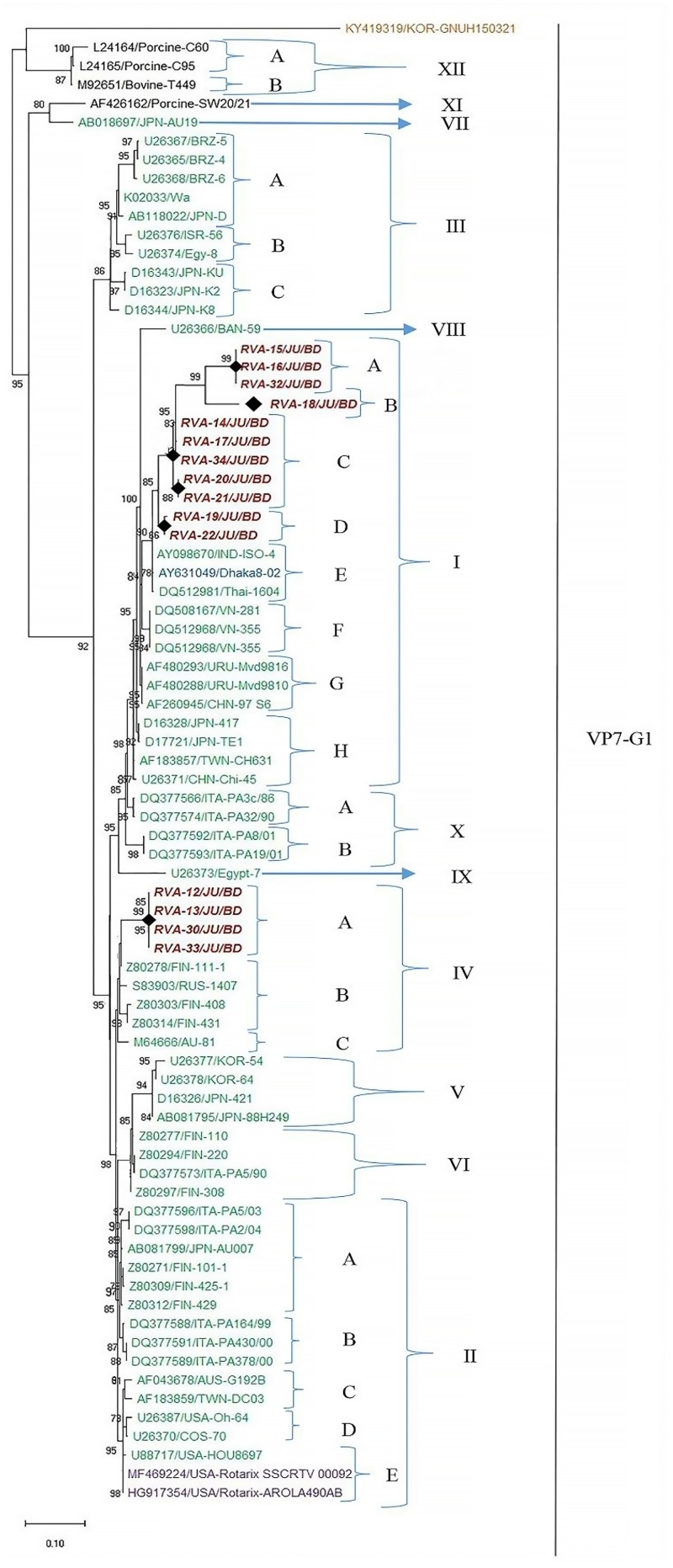
Maximum likelihood phylogenetic tree constructed from the nucleotide sequences of G1-VP7 strains and representative RVA strains with kimura-2-parameter model in MEGA X. Lineages analysis was conducted using reference trees. Bootstrap values <60 are not shown. RVA strains sequenced in this study are represented by the red color in italic bold letter. Bangladeshi strains reported in the previous studies are shown in blue. The vaccine strains are represented by purple color, while green shading represent strains isolated all over the world and black color represented nonhuman primate RVA strains. The RVA strains sequenced in this study are represented by their ID and reference strains obtained from GenBank database are represented by accession number and Strain Name. Scale bar: 0.10 substitutions per nucleotide.

The sequences of VP7 genes of G1 isolates detected in this study and worldwide rotavirus reference G1 strains were used to investigate the heterogeneity of rotaviruses within G1 genotype. Twelve distinct lineages and **23** sublineages were identified (Fig 8). Among these, lineage XI and lineage XII consisted of nonhuman primate rotaviruses, while the lineage I to X were found only in humans. Fifteen sequences of G1 genotype from Bangladesh during 2014–2019 were classified into two distinct lineages, lineage I with 73.3% (11 of 15) isolates and the lineage IV with 30.77% (4 of 15) isolates. Within the lineage I, three isolates clustered in sublineage IA, one isolate in sublineage IB, five isolates in sublineage IC and two isolates in ID. G1 isolates of lineage I were closely related with each other and previously isolated G1 of Bangladesh and India. Within the lineage IV, four G1 isolates of this study made a unique sublineage (IVA) (Fig 8). G1 isolates of lineage IV were closely related with G1 rotavirus of Finland and Russia. Within each sublineage, the nucleotide identities of rotavirus strains ranged from 97% to 100%, indicating less than 3% genetic difference among them. On the contrary, the nucleotide sequence variation of rotavirus G1 strains between lineages was higher, ranging from 6% to 12%.

### Mutation analysis of VP7 gene of group A rotavirus

Rotavirus VP7 protein has three important antigenic regions (region A—amino acid position 87 to 100, region B—amino acid position 141–150, and region C—amino acid position 208–224), which can be variable within genotype. About 90%-99% sequence similarities were found in the genotype G1P[8] of this study with vaccine strain MF469224 and JN849114. In five G1P[8] strains, substitution point mutations were detected in the region A. However, all G2P[4] strains had the same mutation G216R at the region C comparing with vaccine strain GU565068. About 95%-99% sequence similarity was detected among G2P[4] strains. No significant mutation was found for G9P[4] strains in the regions A, B or C. Two G1P[6] strains had a deletion mutation at the position 220. G9P[8] strains had several mutations at regions A and C, sharing 95% sequence similarity. G9P[6] strains had substitution mutation at the regions B and C, sharing 96% sequence similarity. Isolate of G11P[25] RVA had only one mutation at the position T97I, sharing 100% sequence similarity with the reference (Table 3).

**Table 3:** Amino acid substitution mutations in the antigenic regions of VP7 in Bangladeshi rotavirus strains detected during 2014–2019.

Genotype (Strain)	Antigenic regions of VP7 [Position (Initial a.a.→ Mutated a.a.)		
	A (87–100)	B (141–150)	C (208–224)
G1P[8] (***RVA-15***)	H88Q	-	-
G1P[8] (***RVA-16***)	C92G	-	-
G1P[8] (***RVA-18***)	I87F, I94R, Q95P, V99E	-	-
G1P[8] (***RVA-23***)	Y87A, R89V, N91S	-	-
G1P[8] (***RVA-32***)	Q95H, L96S, L100F	-	-
G2P[4] (***RVA-1***)	-	-	T218N
G2P[4] (***RVA-2***)	-	-	T218N
G2P[4] (***RVA-9***)	-	-	T218N
G9P[8] (***RVA-28***)	Q96H, T97S, G100V	-	N210Y
			K224N
G9P[6] (***RVA-29***)	-	A147P	N215H, C224R
		N149H	
		I150K	
G11P[25] (***RVA-31***)	T97I	-	-

^a.a.^ amino acid.

- no mutation.

## Discussion

Still in this 21^st^ century rotavirus infection is a burden for children in developing countries like Bangladesh. Virus infection in the gastrointestinal tract is one of the major causes of children morbidity and mortality in developing countries [1, 2, 4]. In agreement with previous studies, this study detected 36.8% (211 of 574) prevalence of virus infection in children with acute gastroenteritis in Bangladesh [19]. It was also found that rotavirus (RV) had the highest prevalence (24.4%), followed by norovirus (4.9%), adenovirus (4.2%) and human bocavirus (3.3%) [25]. Prevalence of viral infection in diarrheal children with AGE reported in this study were similar with the previous studies in developing countries like Nepal, India, Sri Lanka, Pakistan, South America, Africa and Europe [19–21, 29–33]. In this study, rotavirus A was detected in 24.4% fecal specimens. No RVB or RVC was detected in this study from Bangladeshi children. This high prevalence of RVA in children with AGE in Bangladesh is an indication of lower vaccine efficacy or poor vaccine coverage. This finding of high RVA prevalence in children was consistent with the previous studies during pre-vaccinated era of rotavirus in Bangladesh and other developing countries [19–21, 29–33]. Other possible causes of high prevalence of RVA might be poor hygienic conditions of most of the residents, presence of great genotypic diversity and not including vaccine in the national immunization program in Bangladesh [19]. In this study, the coinfection of other viruses with rotavirus was also determined. Coinfection between diarrheal viruses was detected in 24.6% of the positive cases. Coinfection between rotavirus and norovirus accounted for most of the cases (10 of 52) of coinfection. These findings are in good agreement with the previous studies of rotavirus coinfection in many developing countries worldwide [33–35].

Children aged <60 months were divided into six age groups according to previous study [25, 33]. In this study, rotavirus was detected to be most prevalent (37.9%) among children aged 4–11 months, followed by 12–23 months (22.1%). Most of the rotavirus positive cases (62.1%) were detected in male children. Both the age and gender distribution of rotavirus in diarrheal children in Bangladesh were similar with the previous epidemiological studies in Bangladesh, Japan, Asia, Europe and South America [20, 29–35]. Rotavirus infection has been reported to cause various clinical symptoms in diarrheal children worldwide. In this study, diarrhea (90.7%) dehydration (88.7%) and vomiting was found in higher percentage in rotavirus positive cases than negative cases. However, abdominal pain was reported in higher percentage from rotavirus negative cases. Clinical symptoms associated with rotavirus infection reported in this study also agreed with the previous studies in Bangladesh, Asia, Africa and Europe [20, 21, 29–33].

While analyzing RVA infection seasonality, we found high incidence of rotavirus during the winter (Nov-Jan) season with low temperature in Bangladesh. About 58.6% of rotavirus infection in children were detected during the winter during 2014 to 2019. Another peak of rotavirus infection (30.7%) was found during the rainy season (May-Jul). During this study, the incidence of rotavirus increased with declining temperature and we found highest peak (21% infection) at 15°C average temperature in Bangladesh. High prevalence and peak of RVA infection had been reported in Dey et al. (2009) and Phan et al. (2007). The seasonality of RVA in this study is with good agreement with studies in Bangladesh, South East Asia and Japan [20, 36, 37].

One of the major objectives of this study was to characterize the genotype of circulating rotavirus in Bangladesh. Changing pattern of both G and P genotypes along with their combinations were characterized. Worldwide epidemiological research on rotavirus specified that G1 is the predominant genotype. However, incidence of genotype G2-G4, G9 and G12 has also been detected over time [14, 28]. During 1992 to 1997, rotavirus G4 genotype was the most common in Dhaka but became less common over time. During 2002–2005, genotype G1 was the most prevalent, while in 2005–2006, G2 was prevalent over G1 in Bangladesh [18–20]. Genotype G9 became predominant during 2006 to 2012 along with G1 in Bangladesh [19]. We found increased frequency of G1 genotype (57%) followed by G2 (20%), G9 (20%) and G11 (2.9%), respectively in children with acute gastroenteritis in Bangladesh. However, the frequency of G1 was found reducing in Giri et al. (2018) in developing countries of Asia. Interestingly, this study reported decreased frequency of G9 and increased frequency of G2 from previous studies, Mahmud-Al-Rafat et al. (2018) in Bangladesh [19]. Of note, G11 was also specified in this study. RVA studies, Giri et al. (2018) and Sadiq et al. (2019) have reported that prevalence of G3 has increased than before in developing countries in Asia and Europe in recent time [33]. However, genotype G3, G4 along with G12 was not detected in this study. Increased prevalence of G1 is in good agreement with previous reports but absence of G3, G4 and decreased prevalence of G9 along with increased incidence of G2 and G11 are new in Bangladesh [18–21]. This altered trends of RVA genotype distribution may also reflect regional variation in recent times. In this study, four P genotypes- P[8], P[4], P[6] and P[25] were detected. Genotype P[8] was the most frequent (45.7%) followed by P[4] (30%), P[6] (14.3%) and P[25] (2.9%) and 7.1% was nontypeable. Among P genotype, P[8] (80%) has remained the most prevalent genotype followed by P[4] and other non-P[8] genotype (20%) in previous studies in Bangladesh, India, Nepal and Pakistan [20, 28]. Of note, a decrease of P[8] frequency was replaced by an increase frequency of non-P[8] genotypes in Bangladesh during 2014–2019. To the best of our knowledge, this will be the first report of genotype P[6] in Bangladesh. There are no recent reports of P[6] from Bangladesh, but it has been reported from Nepal and Myanmar in Giri et al. (2018).

In the G and P genotype combination, G1P[8] was predominant (22.4%), followed by G9P[8] 20.8%, G2P[4] 16.9%, and G12P[8] 10.4% during 2006 to 2012 in Bangladesh. Further, genotype G3P[8] and G4P[8] had been also reported in Bangladesh [19, 35]. Another unusual genotype G9P[4] has emerged in Bangladesh in recent time and this genotype has also been reported in Asia, Europe, Africa and Americas [19, 29–33]. However, we also detected a great genotypic diversity of RVA during 2014–2019 in Bangladesh. Seven genotype combinations were revealed in this study. The most prevalent genotype was G1P[8] with the highest frequency of 43%, followed by G2P[4] (18%). This genotypic distribution of RVA in Bangladesh was in good agreement with the previous reports in India, Nepal, Pakistan, Japan, Brazil, Europe, Africa and Bangladesh [18–20, 23, 28–33]. Interestingly, we found that the frequency of previously predominant G9P[8] infection has reduced to 3%. We also detected high prevalence (12%) of genotype G9P[4], followed by genotype G1P[6] (11.4%), G9P[6] (3%), and G11P[25] (3%). To the best of our knowledge, it is the first report of G1P[6] and G9P[6] in Bangladesh. There are limited studies that had reported G1P[6] and G9P[6] in Nepal and Myanmar during 2009–2015 and Sadiq et al. (2019) has reported G1P[6] and G9P[6] in Pakistan [28]. True genotypic diversity of RVA in developing countries like Bangladesh may be greater than we can detect. In future, more studies should be conducted to measure the actual burden of RVA in Bangladesh.

Based on the divergence of VP7 genes of G1 rotaviruses a classification scheme has been developed that specifies G1 rotavirus into various lineages and sublineages. Numerous studies had been undertaken to specify the heterogeneity and dynamics of the evolution of G1 rotaviruses in USA, Italy and Japan [23, 38, 39]. In this study, we followed the classification scheme that was used in Phan et al. (2007) and Dey et al. (2009) to characterize the G1 rotavirus in Bangladesh. The sequences of VP7 gene of G1 genotype were analyzed to investigate the heterogeneity of G1 rotavirus in Bangladesh during 2014–2019. Among twelve lineages, Bangladeshi G1 RVA were distributed in two lineages. Within lineage I, VP7 of G1 from Bangladesh had 99% sequence similarity with IND-ISO-4 and Dhaka8-02 strains. Further, four G1 within lineage IV had 99.98% sequence similarity with FIN-408, FIN-431, AU-81 and FIN-111-1. We detected close relationship between G2P[4] and previously reported M315, M334 and M313 in Bangladesh. Both G1P[8] and G2P[4] genotypes in Bangladesh showed close relationship with previously isolated strains in Bangladesh. Our study is an important tool to understand the genetic diversity of circulating rotavirus in children with AGE in Bangladesh. The presence of diverse genotypes with mutation in antigenic regions can cause reinfection in vaccinated population and reduction of vaccine efficiency in Bangladesh. Further, circulation of diverse genotypes is the major reason of high prevalence of rotavirus in Bangladesh [17, 40].

Rotavirus VP7 protein has three important antigenic regions- amino acid position 87 to 100 (A), 141–150 (B) and 208–224 (C) which are involved in interaction with antibodies and neutralization of epitopes [41, 42]. We detected mutations at antigenic determination regions in G1P[8] and G2P[4] in Bangladesh during 2014–2019. Mutations at antigenic regions may alter rotavirus antigenic properties [41, 42]. Prevalence of unusual genotypes, intragenotypic diversity and frequent mutations at antigenic regions of rotavirus A are major explanations of the constant predominance of rotavirus in Bangladesh.

Bangladesh is one of the countries with the highest disease burden of RVA infections in children <5 years of age. The introduction of RVA vaccines has reduced the global disease burden of RVA in many countries [29]. According to recent survey by Mahmud-Al-Rafat et al. (2018), RVA vaccine coverage in capital Dhaka is good but in small town and suburban areas the RVA vaccine coverage are poor. Overall, RVA vaccine introduction in EPI program in Bangladesh could prevent over 10,000 deaths per year [19]. Besides, RVA studies including large number of samples for sustained time periods and diverse sentinel sites throughout the country will be required for assessing the true RVA disease burden. Moreover, whole genome analysis of human RVA strains and inclusion of animal and environmental samples in future studies is recommended to elucidate the current interspecies transmission and genomic reassortment and recombination events.

## Conclusions

This study concludes the high prevalence of G1P[8] and G2P[4] RVA strains along with reduced prevalence of G3 RVA in Bangladesh during 2014–2019. This study will provide comprehensive insights to the researchers and public health authorities to measure the RVA disease burden in the country. The observed large diversity of RVA genotypes along with their yearly and seasonal fluctuations require an in-depth, broad surveillance system in the country. The findings of this study can provide guideline data before introduction of rotavirus vaccine in the national immunization program in Bangladesh. The data in this study can be used to assess the impact of rotavirus vaccine in the future.

## Supporting information

S1 Chart GenBank accession numbers of rotavirus strains used in this study based on VP7 sequences. (DOCX)
