# Supplementary material for: Molecular epidemiology and surveillance of circulating rotavirus among children with gastroenteritis in Bangladesh during 2014–2019
Source: PLoS One. 2020 Nov 30;15(11):e0242813. doi: 10.1371/journal.pone.0242813 (PMC7703916; doi:10.1371/journal.pone.0242813)
Supplement: S1 Chart — (DOCX) [file pone.0242813.s001.docx]

**Reference strains used in this study for VP7-G1 strains:**

JX411971 (MP-GWL), FJ348350 (Ha95), FJ348348 (Ha45), FJ435209 (Nov08-3427), GU390461 (Nov07-2523), JX841120 (Nov07-2058), MK852665 (IDH 7364), MK852661 (8494), MK852650 (IDH 7278), MK852644 (IDH 7243), MK852641 (IDH 7227), MK050140 (3000503711), MK050136 (176), MK050135 (168), MF673439, MN758631 (30), MN758630 (17), KX681837 (NIH-BBH3988), JX027905 (CK00092), HQ738627 (Omsk08-423), MH191272 (PAK86), KY497545 (PAK/42), KJ753754 (MRC-DPRU1269), KP007144 (TGE12-045), HQ392371 (BE00042), JN849114 (Rotarix-A41CB052A), MG571803 (VME8A), KY616899 (JP11786), MF469224 (Rotarix_SSCRTV_00092), HG917354 (Rotarix-AROLA490AB), LC028930 (OSN9-Rx), KC580617 (DC3828), KY616907 (JP12729), MN549967 (BS5d), KJ626529 (PRY/404) , KP883198 (Mali-137), DQ492674 (Dhaka16), AY631049 (Dhaka8-02) , U26378 (Kor-64), D16326 (421), HQ650124 (DS-1).

**Reference strains used in this study for VP7-G2 strains:**

KU356601 (BGN/M315), KU199286 (BGN/M334), KU356612 (BGN/M313), KP752805 (MRC-DPRU308), KP752665 (MRC-DPRU295), KC443789 (CK20051), KC442897 (2008747095), JX965170 (AUS/WAPC703), JX965169 (AUS/WAPC681), KF648941 (RUS/S12-14), KF006862 (RUS/O1457), MH277406 (PAK347), MH557059 (NIH-BBH-4684), MN577199 (NS18-A1455) , MN577198 (NS18-A1454), MH557060 (NIH-BBH-4705), KY497534 (PAK/3085), KR705272 (BEL/BE34), KF812584 (Seoul1602) KF812583 (Seoul1433) KF202499 (ITA/BERG08), JQ069521 (CAN/RT008-09), KX574260 (RV1112), KP007170 (/RVA/Human-PHI/TGO12-007), KP007148 (PHI/TGO12-003), EF690795 (Dhaka26-06), KX352367 (BRA/RV100104-RR), KJ638622 (BRA/RS15633), KR705415 (BEL/BE85) , EF690797 (Matlab6-04), MH382856 (BD522), AB831009 (NPL/JS126), AB830966 (NPL/04N596), MH382858 (ETH/BD526), KU356590 (BGN/J251), KC442979 (USA/VU08-09-38), EF690796 (BGD/Dhaka39-03), EF690793 (BGD/Dhaka16-04), EF690786 (BGD/Mathbaria2), KP882186 (BGD/Bang-114), KP881922 (BGD/Bang-016), LC482501 (JPN/S2), LC228364 (JPN/K-21-16) LC228353 (JPN/K-3-16), LC228342 (JPN/CH1020), GU565068 (RotaTeq-SC2-9), U73955 (USA/95A), JF304920 (KEN/D205), AY261347 (906SB), KT694944 (USA-Wa).

**Reference strains used in this study for VP7-G9 strains:**

LC227990 (Kol-040), LC227989 (Kol-028), LC227991 (Kol-041), LC227996 (Kol-065), LC227993 (Kol-051), LC227987 (Kol-006), MN067177 (CMC_00006), MN066996 (CMC_00005), LC172413 (JPN/SP029), LC172405 (JPN/SP021), LC172400 (JPN/SP014), LC172390 (JPN/OT039), LC172389 (JPN/OT038), FJ447573 (Nov08-3428) , HQ445974 (Nov08-3379), FJ529389 (Nov05-237), GU377146 (Nov05-114), LC514541 (DBM2017-014), LC514475 (DBM2017-016), MH182443 (PAK56), AY307094 (Perth-G9) , AY307087 (Melb-G9.10), LC514552 (DBM2018-111), JX195068 (ITA/AV21), MH591311 (LBNN469) , MH591306 (LBNN418), KP882208 (Bang-116), KP882065 (Bang-081), KP882043 (Bang-079), KM042984 (BR6-05), KJ751987 (MRC-DPRU1734), KF636305 (MRC-DPRU1708), JN013998 (2371WC), KJ753134 (MRC-DPRU2343), KJ752656 (MRC-DPRU1741), KJ752612 (MRC-DPRU2229), KP222836 (MOZ/21162), AB905466 (BTN-88), KY419318 (GNUH150305) KY419310 (GNUH150309), KY419306 (GNUH1504160), LC437649 (NPL-TK279), KY497491(PAK-HF66), LC437653 (NPL-TK2554), KX265695 (EGY-AS997), LC482504 (USA-WI61), L14072 (IND-116E), JF521480 (USA-DC/G2275).

**Reference strains used in this study for VP7-G11 strains:**

HQ198807 (KOR-CAU-1), EU259896 (KOR-GJ0703034), EF121951 (KOR), GQ149096 (EC2184), LC433801 (NPL-TK2615), GU199497 (NPL-KTM368), AY773003 (Dhaka6), GU199508 (Matlab36), KC140587 (CAU12-2), EF033338 (BGD-Matlab36), JX040425 (IND-N-38), KX363350 (Pig-VNM-14150_54), DQ482712 (Dhaka22), DQ482718 (Dhaka13) , MN203538 (Pig-SVK-1CICIAK), GQ149096 (EC2184), KF614051 (Pig-BEL-12R021) , KT727271 (Pig-CMP-002-13).

**Reference (Accession no.) strains used in this study for G1 genotype lineage analysis**:

(L24164) Porcine, (L24165) Porcine, (M92651) Bovine, (AF426162) Porcine, (AB018697) JPN, (U26378) KOR-64, (U26377) KOR-54, (D16326) JPN-421, (AB081795) JPN-88H249, (U26366) BAN-59, (Z80303) FIN-408, (Z80314) FIN-431, (M64666) AU-81, (DQ377596) ITA-PA5/03, (DQ377598) ITA-PA2/04, (DQ377588) ITA-PA164/99, (DQ377591) ITA-PA430/00, (DQ377589) ITA-PA378/00, (Z80312) FIN-429, (AB081799) JPN-AU007, (Z80271) FIN-101-1, (Z80309) FIN-425-1, (AF043678) AUS-G192B, (AF183859) TWN-DC03, (U26387) USA-Oh-64, (U26370) COS-70, (Z80277) FIN-110, (Z80294) FIN-220, (Z80297) FIN-308, (DQ377573) ITA-PA5/90, (DQ377566) ITA-PA3c/86, (DQ377574) ITA-PA32/90, (DQ512981) Thai-1604, (AY631049) Dhaka8-02, (DQ508167) VN-281, (DQ512968) VN-355, (DQ512968) VN-355, (AF480293) URU-Mvd9816, (AF480288) URU-Mvd9810, (AF260945) CHN-97'S6, (AF183857) TWN-CH631, (AY098670) IND-ISO-4, (U26371) CHN-Chi-45, (D16328) JPN-417, (D17721) JPN-TE1, (U26373) Egypt-7, (K02033) Wa, (U26374) Egy-8, (DQ377592) ITA-PA8/01, (DQ377593) ITA-PA19/01, (U26368) BRZ-6, (U26367) BRZ-5, (U26365) BRZ-4, (U26376) ISR-56, (D16343) JPN-KU, (D16323) JPN-K2, (AB118022) JPN-D, (D16344) JPN-K8, (S83903) RUS-1407, (U88717), (KY419319) KOR-GNUH150321.
